# Editorial: Control of Plant Pathogens by Biogenic Elicitors and Possible Mechanisms of Action

**DOI:** 10.3389/fpls.2016.00340

**Published:** 2016-03-24

**Authors:** Vitaly G. Dzhavakhiya, Larisa A. Shcherbakova

**Affiliations:** ^1^Department of Molecular Biology, All-Russian Research Institute of PhytopathologyMoscow, Russia; ^2^Laboratory of Physiological Plant Pathology, All-Russian Research Institute of PhytopathologyMoscow, Russia

**Keywords:** biogenic elicitors, biocontrol, plant defense mechanisms, defense-related genes, systemic resistance, priming, apoplast, crop protection

Elicitors of biological origin, especially those that are able to control plant pathogens by induction of plant systemic resistance are being intensively investigated because of their great potential for crop protection and environmental compatibility. Since the range of induced defense responses can depend on the elicitor used, the plant treated and the target pathogen, a detailed study of the modes of action of biogenic elicitors is required prior to their use.

In this context, an important aim of the Research Topic on *Control of plant pathogens by biogenic elicitors and possible mechanisms of action* was a better understanding of the mechanisms underlying the protective effect of microorganisms with a potential or already established role in biocontrol. Interestingly, works included in the topic report that a biocontrol agent is simultaneously able to activate both SAR and ISR defense pathways in the same plant (e.g., Salas-Marina et al.; Song et al.). Thus, tomato root colonization with the arbuscular mycorrhizal fungus *Funneliformis mosseae* induced a range of plant defense reactions, and primed defense responses, including the activation of some enzymes and the up-regulation of *PR* genes associated with SAR upon challenge with *Alternaria solani*. At the same time, it was also shown that JA-dependent signaling was necessary for mycorrhiza-primed systemic resistance to this pathogen (Song et al.).

Plant disease resistance is triggered by various elicitors, which are widely presented in pathogenic and non-pathogenic fungi, bacteria and oomycetes. Biogenic elicitors (glycoproteins, lipids, or oligosaccharides) as well as microbial proteins and peptides triggering defense responses play an important role in the development of local and systemic resistance. In the current research topic, several publications confirm the importance of such proteins for plant-mediated interactions of biocontrol fungi with soil or foliar pathogens of various life styles. They report both common features and individual specificity concerning the mechanisms of action of various proteinaceous elicitors. For instance, colonization of plant roots with saprophytic *Fusarium oxysporum* or *Trichoderma* strains protects tomato against vascular wilt and other diseases via distinct mechanisms including induction of local and systemic resistance. Proteins, such as CS20EP produced by *F. oxysporum* strain CS-20 (Shcherbakova et al.) as well as Sm1 and Epl1 from *Trichoderma* spp. (Salas-Marina et al.) strongly contribute to the activation of defense-responsive genes in the pretreated plants. Experiments on systemic tomato protection with wild, e*pl1*- and *sm1*-deletion or overexpression strains against necrotrophic (*A. solani, Botrytis cinerea*) and hemibiotrophic (*Pseudomonas syringae* pv. *tomato*) pathogens indicate that Sm1 and Epl1 induce both ISR and SAR, affecting different specific targets in the same pathway depending on the life style of the pathogen (Salas-Marina et al.). CS20EP identified as a new fungal small cysteine-rich protein is considered as a likely candidate for elicitation of the ion exchange response in plant cells and enhanced *PR-1* expression, both of which may result in a mitigation of *Fusarium* wilt severity on tomato seedlings (Shcherbakova et al.). Another research related to protein- and peptide-containing elicitors and their possible mode of action (Nesler et al.) focuses on studying the applicability of such elicitors under field conditions. The authors show that a natural derivative from meat and yeast effectively controls powdery mildew on grapevine in field, and provide data that the crop protection level is comparable to the level achieved after plant treatment with sulfur used as a standard fungicide. Importantly, expression of grapevine defense-related genes induced with the tested elicitor was observed not only prior to infection but also at early stages of the disease, and the protection was effective over the period of three-year field trials (Nesler et al.).

Li and colleagues presented the work on identification and functional study of the velvet gene *FocVel1* in the cucumber wilt pathogen (*F*. *oxysporum* f. sp. *cucumerinum*), which disruption reduces the fungal pathogenicity, growth and reproduction (Li et al.).

The apoplast is the first line of defense, where various scenarios of the plant-microbe battle take place. Saprotrophic or pathogenic microorganisms can induce alterations in the biochemical pattern of apoplastic metabolites, thereby influencing the outcome of relationships with host or non-host plants. Research by Baker and co-workers covers some aspect of plant-microbe interactions in the apoplast, viz. redox/phenolic events, which are induced by pseudomonads. The authors report that tobacco plants respond to saprophytic, virulent or avirulent *Pseudomonas* bacteria by different changes in apoplastic phenolics. Some of these changes are specifically caused by pathogen infection and suggest weakening of the apoplast/symplast barrier (Baker et al.).

New findings about mechanisms resulting in basal resistance against root-knot nematodes (RKN) are presented by Zhou and colleagues. They showed that plant inoculation with *Meloidogyne incognita* induced the transcription of JA- and NO-related genes in tomato, and also demonstrated that leaf spraying with JA and NO suppressed nematode development in tomato roots and decreased the negative influence of the pathogen on photosynthesis. According to the authors' suggestion, NO-involving defense against RKN is likely associated with the JA-dependent signaling pathway. The key role of *PI2* (protease inhibitor 2 encoding gene) in tomato resistance to RKN is also emphasized (Zhou et al.).

Collectively, the articles published in the topic confirm a high potential of biogenic elicitors for plant pathogen control, promote in-depth analyses of elicitation mechanisms and evidence that biogenic elicitors may become a powerful tool in the protection of crop plants from diseases.

## Author contributions

All authors listed, have made substantial, direct and intellectual contribution to the work, and approved it for publication.

### Conflict of interest statement

The authors declare that the research was conducted in the absence of any commercial or financial relationships that could be construed as a potential conflict of interest.

